# Mouse LIMR3/CD300f is a negative regulator of the antimicrobial activity of neutrophils

**DOI:** 10.1038/s41598-018-35699-4

**Published:** 2018-11-27

**Authors:** Keigo Ueno, Makoto Urai, Kumi Izawa, Yoshiko Otani, Nao Yanagihara, Michiyo Kataoka, Shogo Takatsuka, Masahiro Abe, Hideki Hasegawa, Kiminori Shimizu, Toshio Kitamura, Jiro Kitaura, Yoshitsugu Miyazaki, Yuki Kinjo

**Affiliations:** 1 Department of Chemotherapy and Mycoses, National Institute of Infectious Diseases, 1-23-1 Toyama, Shinjuku-ku, Tokyo 162-8640 Japan; 2 Atopy Research Center, Juntendo University School of Medicine, 2-1-1 Hongo, Bunkyo-ku, Tokyo 113-8421 Japan; 3 Department of Biological Science and Technology, Faculty of Industrial Science and Technology, Tokyo University of Science, 6-3-1 Niijuku, Katsushika-ku, Tokyo 125-8585 Japan; 4 Department of Pathology, National Institute of Infectious Diseases, 1-23-1 Toyama, Shinjuku-ku, Tokyo 162-8640 Japan; 5 Division of Cellular Therapy, Advanced Clinical Research Center, The Institute of Medical Science, The University of Tokyo; Division of Stem Cell Signaling, The Institute of Medical Science, The University of Tokyo, 4-6-1 Shirokanedai, Minato-ku, Tokyo 108-8639 Japan; 6 Present Address: Department of Chemistry for Life Sciences and Agriculture, Faculty of Life Sciences, Tokyo University of Agriculture, 1-1-1 Sakuragaoka, Setagaya-ku, Tokyo 156-8502 Japan; 7 Present Address: Department of Bacteriology, The Jikei University School of Medicine, 3-25-8 Nishi-Shimbashi, Minato-ku, Tokyo 105-8461 Japan

## Abstract

Leukocyte mono-immunoglobulin-like receptor (LMIR)/CD300 proteins comprise a family of immunoglobulin-like receptors that are widely expressed on the immune cell surface in humans and mice. In general, LMIR3/CD300f suppresses the inflammatory response, but it can occasionally promote it. However, the precise roles of LMIR3 in the function of neutrophils remain to be elucidated. In the present study, we investigated LMIR3 expression in mature and immature neutrophils, and evaluated the effects of LMIR3 deficiency in mouse neutrophils. Our results indicated that bone marrow (BM) neutrophils expressed LMIR3 on their cell surface during cell maturation and that surface LMIR3 expression increased in response to *Pseudomonas aeruginosa* infection in a TLR4/MyD88-dependent manner. LMIR3-knockout (KO) neutrophils displayed significantly increased hypochlorous acid production, and elastase release, as well as significantly augmented cytotoxic activity against *P*. *aeruginosa* and *Candida albicans*; meanwhile, inhibitors of elastase and myeloperoxidase offset this enhanced antimicrobial activity. Furthermore, LMIR3-KO mice were significantly more resistant to *Pseudomonas* peritonitis and systemic candidiasis, although this may not be entirely due to the enhanced activity of neutrophils. These results demonstrate that LMIR3/CD300f deficiency augments the antimicrobial activity of mouse neutrophils.

## Introduction

Leukocyte mono-immunoglobulin-like receptor (LMIR)/CD300 proteins are immunoglobulin-like receptors that are widely expressed on the surface of immune cells in humans and mice. Among the LMIR/CD300 proteins, only two receptors, namely LMIR1/CD300a and LMIR3/CD300f (hereafter LMIR3), contain the immunoreceptor tyrosine-based inhibitory motif (ITIM) in the long cytoplasmic domain. When these receptors form ligand–receptor complexes, the tyrosine residues of ITIM are phosphorylated, and they recruit several types of protein phosphatases such as Src homology 2-containing protein tyrosine phosphatase 1 (SHP-1), SHP-2, and SH2-containing inositol 5-phosphatase 1, leading to the transmission of inhibitory signals^1,2^.

Surface LMIR3 expression has been observed in myeloid cells, including dendritic cells (DCs), macrophages (MΦs), granulocytes, and some B cells in mice^1,2^. In mast cells, LMIR3 recognises ceramide and suppresses cytokine production and degranulation induced via FcεRI-mediated stimulation^3^. This ceramide-mediated suppression is reasonable because the plasma ceramide concentration is unregulated during the inflammatory state^4,5^. In addition, a recent study using the cecal ligation and puncture (CLP) mouse model illustrated that the disruption of the ceramide–LMIR3 interaction protected mice against septic peritonitis via enhanced recruitment of neutrophils to the peritoneal cavity^6^. Another study revealed that LMIR3 expressed on the surface of MΦs and DCs recognise phosphatidylserine (PS), which is regarded as an “eat-me” signal for apoptotic cells and is required for the differential regulation of efferocytosis by MΦs and DCs. Although LMIR3-knockout (KO) MΦs do not engulf most apoptotic cells, the efferocytosis function was clearly activated in LMIR3-KO DCs^7,8^. These data indicate that LMIR3 likely acts on both activating and inhibitory receptors. Indeed, the cytoplasmic tail of LMIR3 also recruits several factors, including p85α, Grb2, and FcRγ chains, during immune activation^1,2^. Furthermore, two recent studies illustrated that murine LMIR3 is a functional receptor of the murine norovirus^9,10^. One of these studies demonstrated that murine norovirus levels were not elevated in the faecal pellets of LMIR3-KO mice^9^. Taken together, it is likely that LMIR3 differentially recognises its ligands and regulates cell function depending on the type of cell in which it is expressed.

Although previous reports indicated that neutrophils express surface LMIR3^11,12^, the function and physiological significance of LMIR3 on the neutrophil surface have not yet been completely elucidated. To reveal the function of the neutrophil surface LMIR3, we investigated the expression profiles of LMIR3 in neutrophils and neutrophil-precursors at steady state and upon microbial stimulation. Using C57BL/6 wild-type (WT) mice and LMIR3-KO mice, we analysed the microbicidal functions of neutrophils and performed *in vivo* infection studies using the gram-negative bacterium *Pseudomonas aeruginosa* and the pathogenic fungus *Candida albicans*. This is the first study to demonstrate the LMIR3-mediated regulation of neutrophil antimicrobial activity.

## Results

### LMIR3 is expressed on the surfaces of myelocytes, metamyelocytes, and neutrophils

**Figure 1:**
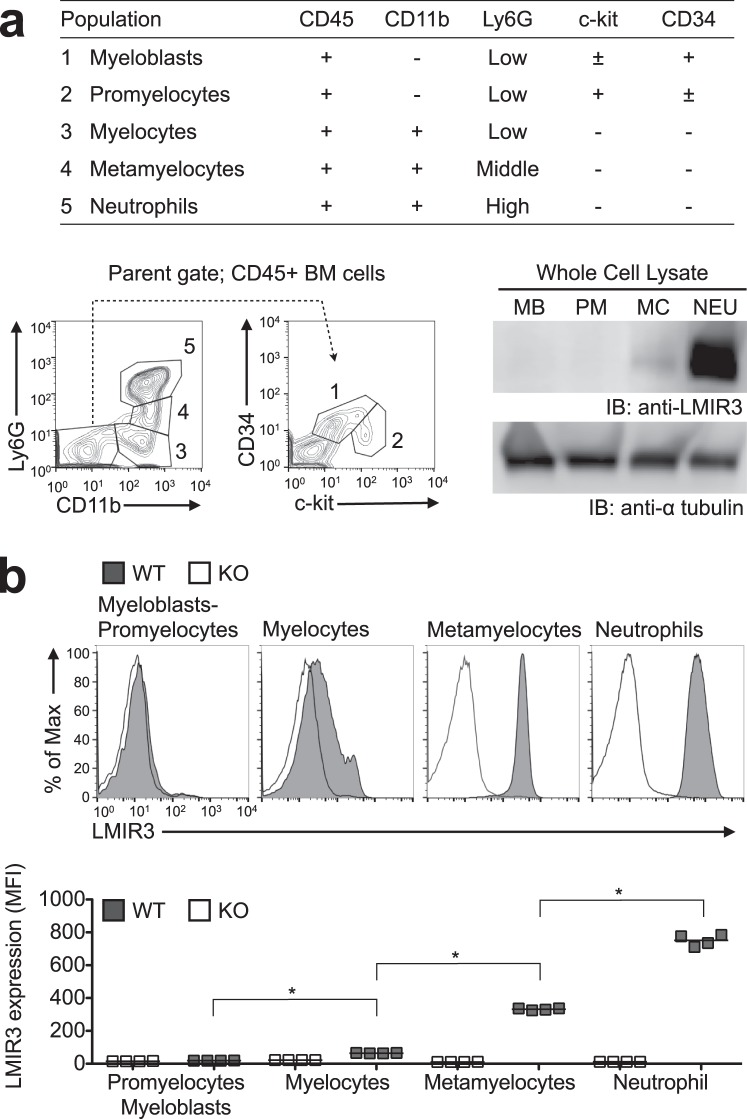
Mouse LMIR3 is expressed on myelocytes, metamyelocytes, and neutrophils. The gating list and flow cytometry profile are shown for the LMIR3 expression analysis. Neutrophils (NEU), myelocytes (MC), promyelocytes (PM), and myeloblasts (MB) in BM were sorted by flow cytometry, and whole-cell lysates were prepared for analysis of the total LMIR3 expression via Western blotting. For the loading control experiment, the membrane was treated with acid–glycine buffer to strip the detection antibodies after immunoblotting for LMIR3, and then α-tubulin was detected on same membrane using a re-probing immunoblot. Full-length blots are shown in Supplementary Fig. 1a. Representative blots from two independent experiments are shown (**a**). Surface LMIR3 expression was evaluated via flow cytometry using the mean fluorescence intensity (MFI). The histogram data and statistical dot plots of MFI are shown. Representative dot plots (n = 4) from two independent experiments are shown. Short horizontal lines indicate average values. *P < 0.05 as determined by analysis of variance with Tukey’s *post hoc* test (**b**).

Previous studies reported surface LMIR3 expression in myeloid cells, including neutrophils^1,2,11^. However, it is unclear at what point during the bone marrow (BM) neutrophil development that LMIR3 appears on the cell surface. We evaluated mouse LMIR3 expression in neutrophils and neutrophil progenitors using immunoblotting and flow cytometry (Fig. 1). Neutrophil progenitors were discriminated using a previously described marker profile^13–15^. Immunoblotting detected LMIR3 expression in the whole-cell lysates of CD11b^+^ Ly6G^−^ myelocytes and CD11b^+^ Ly6G^+^ neutrophils, but not in that of CD45^+^ CD34^+^ c-kit^low^ myeloblasts or CD45^+^ CD34^low^ c-kit^+^ promyelocytes (Fig. 1a and Supplementary Fig. 1a). Surface LMIR3 expression was also detected in myelocytes, metamyelocytes, and neutrophils (Fig. 1b and Supplementary Fig. 1b).

To evaluate human LMIR3 (hLMIR3) expression during neutrophil differentiation, the human promyelocytic cell line HL-60 was differentiated via exposure to dimethyl sulfoxide (DMSO), and surface hLMIR3 expression was measured via flow cytometry. As indicated by the increased CD11b expression, DMSO treatment induced HL-60 differentiation, and the differentiated HL-60 cells expressed hLMIR3 on their surfaces (Supplementary Fig. 1c).

### Bacterial pathogens and Toll-like receptor (TLR) ligands increase LMIR3 expression on the neutrophil surface

**Figure 2:**
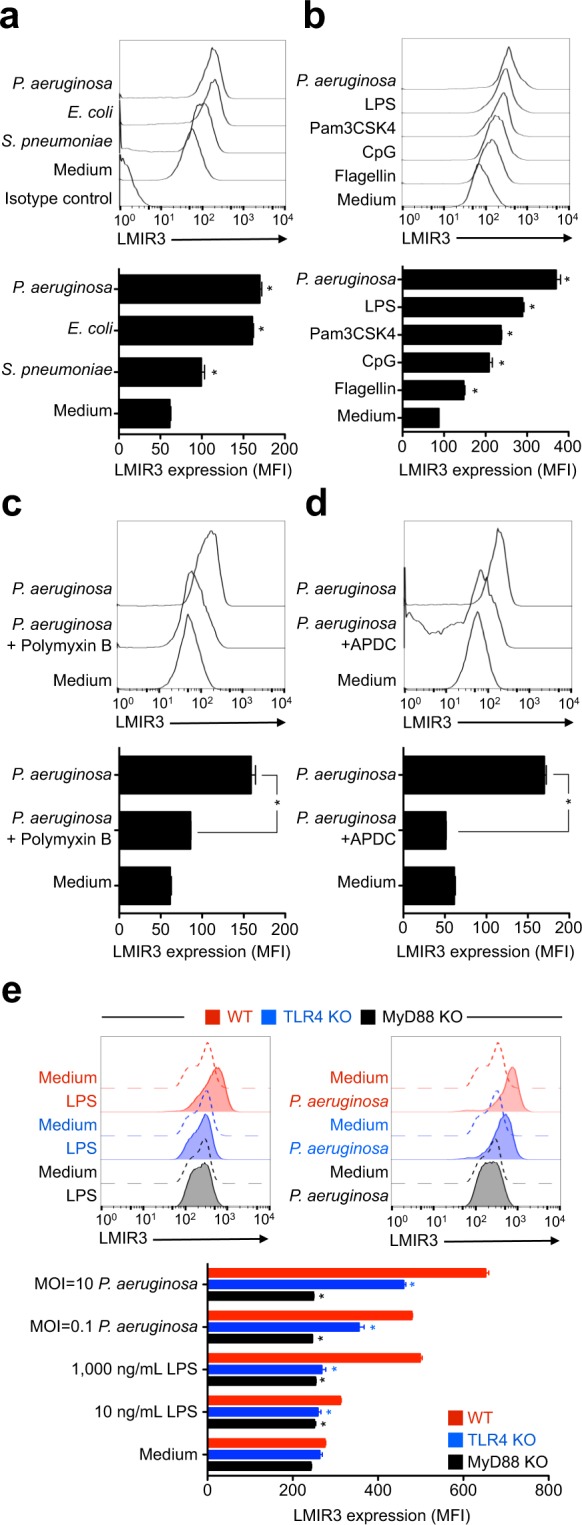
Pathogen-induced surface LMIR3 expression is mediated by the TLR4/MyD88 pathway. (**a**,**b**) Mouse BM cells (3 × 10^6^ cells/mL) were cultivated in triplicate wells for 18 h with gram-negative and gram-positive bacteria (**a**) or TLR ligands (**b**). The ligands and concentration were as follows: heat-inactivated bacteria (multiplicity of infection, MOI = 10), 100 ng/mL flagellin, 5 μg/mL CpG ODN 2216, 100 ng/mL Pam3CSK4, and 1,000 ng/mL LPS, *E*. *coli* serotype R515. The maximum concentration of each TLR ligand was used according to the manufacturer’s instructions. (**c**,**d**) Heat-inactivated *P*. *aeruginosa* strain PAO1 (**c**, MOI = 0.1; **d**, MOI = 10), the LPS inactivator polymyxin B (100 μg/mL), and the NF-κB inhibitor APDC (10 μg/mL) were used. Polymyxin B and heat-inactivated bacteria were premixed and added to the wells containing BM cells. The BM cells were pre-treated for 1 h with APDC, and then heat-inactivated bacteria were added to the wells. (**e**) The surface LMIR3 expression was evaluated for WT, TLR4-KO, and MyD88-KO neutrophils. High-purity, TLR analysis-grade LPS of the *E*. *coli* serotype O55:B5 was used. The cells were analysed via flow cytometry as described above. The flow cytometry gate was set for CD11b^+^ Ly6G^+^ neutrophils. The flow cytometry profile (upper) and bar graph (lower) of the LMIR3 expression are depicted. Representative data (mean ± SDs) from three independent experiments are shown. *P < 0.05 versus medium control (**a**,**b**) and versus WT mice (**e**) by unpaired *t*-test.

It is likely that LMIR3 expression is correlated with the neutrophil maturation/activation status. Consequently, we tested whether LMIR3 expression on neutrophils was further upregulated upon microbial infection and stimulation with TLR ligands (Fig. 2). Microbial stimulation enhanced surface LMIR3 expression on CD11b^+^ Ly6G^+^ neutrophils. In particular, gram-negative bacteria, including *P*. *aeruginosa* and *Escherichia coli*, and lipopolysaccharide (LPS) efficiently increased surface LMIR3 expression (Fig. 2a,b). In the absence of microbial stimulation, LMIR3 expression on neutrophils did not observably change during cultivation (Supplementary Fig. 2a).

Because the TLR4/MyD88/NF-κB pathway is essential for recognising gram-negative bacteria and LPS^16^, we validated the effects of polymyxin B (an LPS inactivator), and ammonium pyrrolidine-1-carbodithioate (APDC, an NF-κB inhibitor) on the enhancement of surface LMIR3 expression upon *P*. *aeruginosa* stimulation. These inhibitors significantly suppressed the enhancement of surface LMIR3 expression on neutrophils stimulated by *P*. *aeruginosa* infection (Fig. 2c,d). Furthermore, the enhancement of surface LMIR3 expression was significantly reduced in TLR4-KO and MyD88-KO mouse neutrophils (Fig. 2e). These results demonstrated that the TLR4/MyD88 pathway was required for the enhancement of surface LMIR3 expression on neutrophils upon infection/stimulation with *P*. *aeruginosa*. Identical behaviour was observed when MACS^®^-enriched Ly6G^+^ neutrophils were used (Supplementary Fig. 2b). This result suggested that other cell types were not required for the upregulation of surface LMIR3 expression on neutrophils (Supplementary Fig. 2b).

**Figure 3:**
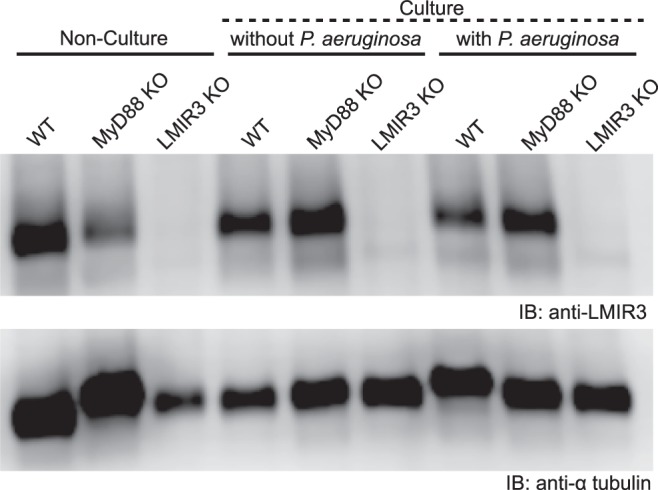
Total LMIR3 expression does not increase after bacterial stimulation. BM cells were cultivated overnight with heat-inactivated *P*. *aeruginosa* (MOI = 10), and then whole-cell lysates were then prepared for immunoblotting analysis. Full-length blots are shown in Supplementary Fig. 2c. For the loading control experiment, the membrane was treated with acid–glycine buffer to strip the detection antibodies after immunoblotting for LMIR3, and then α-tubulin on same membrane was detected with a re-probing immunoblot on the same membrane. Representative blots from three independent experiments are shown.

Next, we tested whether the total LMIR3 expression was upregulated upon stimulation with *P*. *aeruginosa*. BM cells were cultivated overnight in the presence of heat-inactivated *P*. *aeruginosa*, and a whole-cell lysate was prepared for immunoblotting. Our results indicated that the LMIR3 expression did not increase upon stimulation (Fig. 3 and Supplementary Fig. 2c). This result demonstrated that the enhancement of surface LMIR3 expression was not correlated with the total LMIR3 expression level. It also suggested that bacterial stimulation enhanced the change in LMIR3 localisation from the cytosol to the cell surface.

### LMIR3 deficiency increases hypochlorous acid (HOCl) production and elastase release in neutrophils

Increased surface LMIR3 expression was correlated with neutrophil maturation; therefore, we hypothesised that some neutrophil functions were regulated by LMIR3. First, we compared the neutrophil percentage among BM leukocytes from WT and LMIR3-KO mice, but found no significant differences (Supplementary Fig. 3a). This is in agreement with the previous findings in which LMIR3-KO mice exhibited no significant differences in myeloid and lymphoid development compared with WT mice^3,17^.

**Figure 4:**
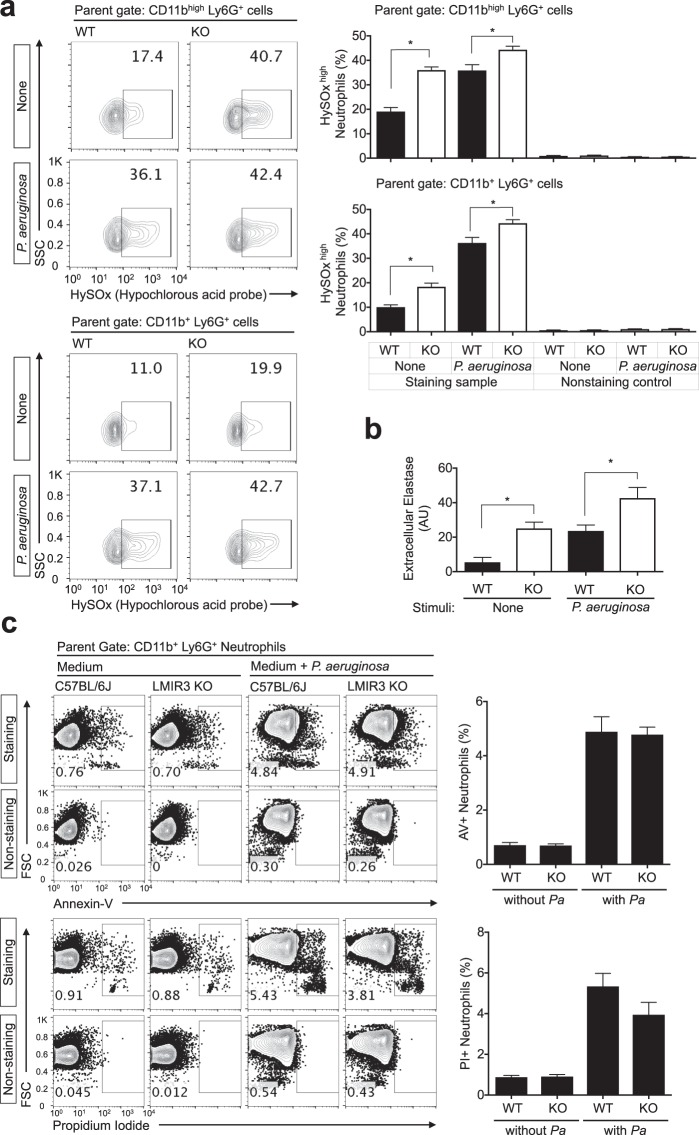
LMIR3 deficiency increases HOCl production and elastase release in neutrophils. (**a**) BM cells were incubated for 1.5 h in HySOx-containing medium with or without heat-inactivated *P*. *aeruginosa* (MOI = 10). The gates were set for CD11b^+^ Ly6G^+^ neutrophils and CD11b^high^ Ly6G^+^ neutrophils, as described in Supplementary Fig. 3a. (**b**) BM cells were incubated for 2 h with or without heat-inactivated *P*. *aeruginosa* (MOI = 10) to release elastase. The elastase activity in the culture supernatants was measured using a fluorescent substrate derived from bovine elastin. The pooled data from two independent experiments are shown (mean ± SEMs, n = 8). *P < 0.05 as determined via an unpaired *t*-test. (**c**) BM cells were incubated for 24 h with or without heat-inactivated *P*. *aeruginosa* (MOI = 10) to detect dead neutrophils. Representative data (mean ± SDs) from three independent experiments are shown. AV, annexin-V; PI, propidium iodide; PA, *P*. *aeruginosa*.

Next, we evaluated the activation status of neutrophils (Fig. 4). HOCl production and elastase release are generally increased in activated neutrophils. HOCl is synthesised from chloride and hydrogen peroxide by myeloperoxidase (MPO) derived from the primary granules. Although HOCl production is enhanced by both phagocytic and non-phagocytic stimuli^18^, neutrophils can basally produce small amounts of HOCl during short-term cultivation in medium in the absence of any stimuli^19^. Furthermore, HOCl plays a pivotal role in antimicrobial activity^20^. In this experiment, we investigated HOCl production via flow cytometry using the HOCl-specific probe HySOx^21–23^. BM cells were incubated for 1.5 h in HySOx-containing medium with or without heat-inactivated *P*. *aeruginosa* and then evaluated via flow cytometry. Our result demonstrated that compared with WT mice, LMIR3-KO mice had a significantly larger population of BM neutrophils that produce high amounts of HOCl after cultivation both with and without stimulation (Fig. 4a). The mean fluorescence intensity (MFI) of HySOx staining was also significantly increased in LMIR3-KO CD11b^high^ Ly6G^+^ neutrophils under the non-stimulatory condition, compared with that in WT neutrophils (Supplementary Fig. 3b). This finding indicated the LMIR3 KO CD11b^high^ Ly6G^+^ neutrophils have enhanced HOCl production. Although primary granules containing elastase can fuse with the phagosomes, these granules also spontaneously undergo exocytosis under non-stimulatory conditions^24^. Elastase activity in culture supernatant was measured with a fluorescent substrate BODIPY-FL-labelled DQ elastin. Our result illustrated that LMIR3-KO BM cells upregulated the elastase activity or releasability in culture supernatants after both unstimulated and stimulated cultivation (Fig. 4b). Previous studies demonstrated that LMIR3 recognises PS on apoptotic cells and that it is involved in the efferocytosis by MΦs and DCs^7,8^. Although we also measured the percentage of dead neutrophils after BM cell cultivation, this value was not increased in LMIR3-KO BM cells (Fig. 4c). This result suggested that the live/dead cell ratio did not explain the elevated HOCl production and elastase release in LMIR3-KO neutrophils. Taken together, these results suggested that LMIR3 deficiency led to neutrophil activation.

### LMIR3 partially regulates the expression of primary granule-related proteins

**Figure 5:**
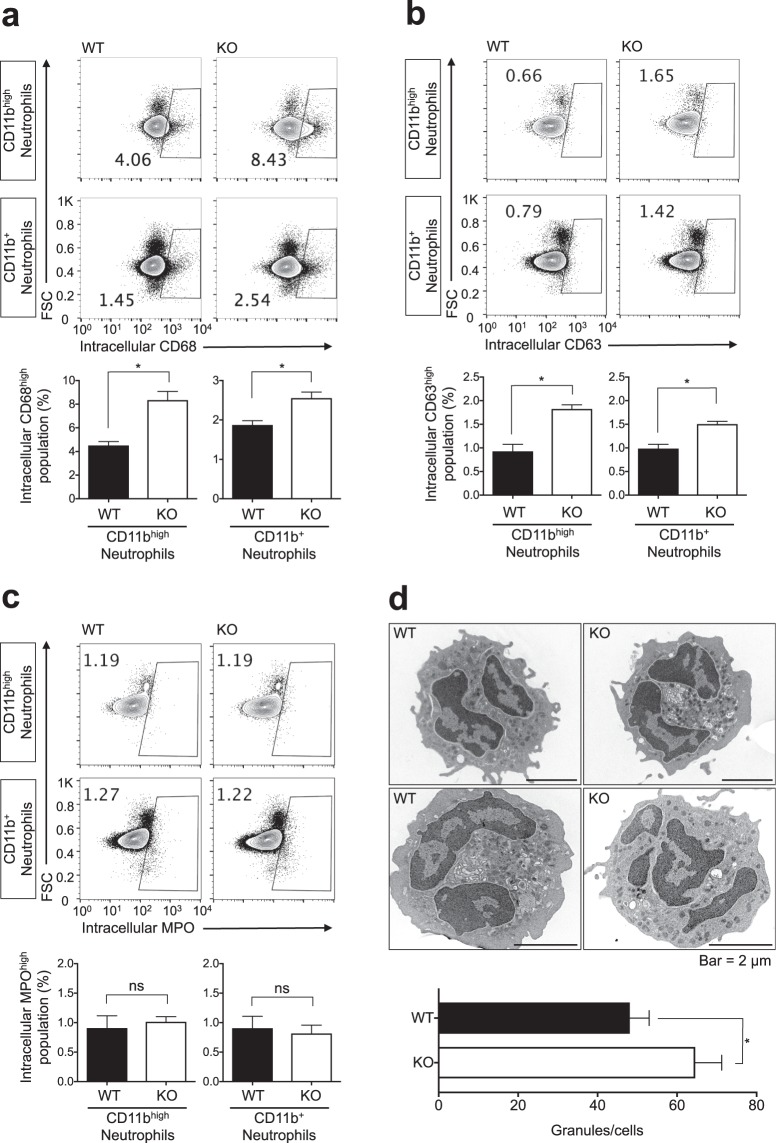
LMIR3 partially regulates the expression of primary granules-related proteins in neutrophils. (**a**–**c**) BM cells were immediately stained and evaluated via flow cytometry after harvesting from the mouse bones. The gates were set for CD11b^+^ Ly6G^+^ neutrophils and CD11b^high^ Ly6G^+^ neutrophils, as described in Supplementary Fig. 3a. Pooled data from two independent experiments are shown (mean ± SEMs, n = 6). *P < 0.05 as determined using an unpaired *t*-test; ns, not significant, (**d**) Histopaque-enriched neutrophils were treated with DAB solution to highlight MPO-containing primary granules and observed via transmission electron microscopy. Nuclear-segmented cells were enumerated as neutrophils. Pooled data from two independent experiments were plotted (mean ± SEMs, n = 20). *P < 0.05 as determined by the Mann–Whitney U test.

Because the content and number of primary granules can affect HOCl production and elastase activity or releasability in culture supernatant, we investigated the expression profile of primary granule-related proteins, including macrosialin (CD68), tetraspanin (CD63), and MPO^25^. Previous transmission electron microscopy analysis revealed that CD68 colocalised with MPO in primary granules^26^, and the expression status of CD68/CD63 was analysed via flow cytometry and immune imaging analysis to evaluate the trafficking of primary granules^27–29^. In this experiment, BM cells were immediately stained and evaluated for intracellular CD68, CD63, and MPO expression via flow cytometry after being harvested from the mouse bones (Fig. 5a). The result showed that all neutrophils clearly expressed these proteins (Supplementary Fig. 3c). Our result demonstrated that LMIR3-KO mice had a slightly larger population of neutrophils expressing high levels of intracellular CD68 than WT mice (Fig. 5a). Similar results were observed for neutrophils with high expression of intracellular CD63, but not MPO (Fig. 5b,c). Furthermore, the number of primary granules in LMIR3-KO segmented neutrophils was slightly increased compared with that in WT neutrophils (Fig. 5d). By contrast, intracellular CD11b expression was comparable between WT and KO mice (Supplementary Fig. 3d). CD11b is localises on the membranes of specific granules, gelatinase granules, and secretory vesicles^25^. These results indicate that LMIR3 partially regulates the expression of primary granule-related proteins in neutrophils. However, these minor LMIR3-KO-mediated changes may not be entirely responsible for the observed high HOCl production and elastase activity or releasability observed.

### LMIR3-KO neutrophils exhibit enhanced antimicrobial activity against bacterial and fungal pathogens

**Figure 6:**
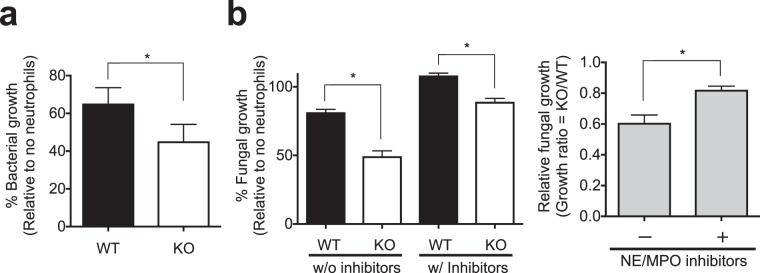
Microbicidal activity is augmented in LMIR3-KO neutrophils. (**a**) Histopaque-enriched neutrophils were cultivated in triplicate wells with *P*. *aeruginosa* (MOI = 0.1) for 1 h, and the suspensions were plated to evaluate the bacterial number. The bacterial growth was calculated as a percentage based on viable counts (colony-forming units, CFU/mL) relative to the counts of the no-neutrophil controls. Representative graphs (mean ± SDs) and images from three independent experiments are shown. *P < 0.05 using an unpaired *t*-test. (**b**) A similar experiment was performed using *C*. *albicans* (MOI = 0.05), the neutrophil elastase (NE) inhibitor Sivelestat, and the MPO inhibitor 4-ABAH. After 24 h of cultivation, the turbidity was measured to evaluate the fungal growth. The fungal growth was calculated as a percentage based on the optical density relative to the counts of the no-neutrophil controls, and the relative fungal growth was also calculated under each condition as compared the findings in WT and KO-neutrophils. Pooled data from three independent experiments were plotted (mean ± SEMs). *P < 0.05 by an unpaired *t*-test.

MPO and elastase from neutrophil primary granules are required to kill bacterial and fungal pathogens, including *P*. *aeruginosa* and *C*. *albicans*^20,30–34^. Therefore, we inferred that the high HOCl and elastase production of LMIR3-KO neutrophils contributes to their bactericidal and fungicidal activities. In this experiment, Histopaque-enriched neutrophils were used to measure antimicrobial activity. An elastase inhibitor (Sivelestat) and a MPO inhibitor [4-aminobenzoic acid hydrazide (4-ABAH)] were also used to evaluate the contribution of both enzymes, as described previously^35,36^. LMIR3-KO neutrophils significantly suppressed the *in vitro* growth of *P*. *aeruginosa* and *C*. *albicans* compared with WT neutrophils (Fig. 6a,b). In addition, the combination of elastase and MPO inhibitors offset the augmented antifungal activity of LMIR3-KO (Fig. 6b). However, these inhibitors did not entirely eliminate the antimicrobial activity of LMIR3-KO neutrophils (Fig. 6b); thus, another pathway conferring antimicrobial activity may also be upregulated in LMIR3-KO neutrophils. This result suggested that LMIR3 deficiency led to the enhanced antimicrobial activity of neutrophils.

### LMIR3-KO mice are more resistant to bacterial and fungal infection

**Figure 7:**
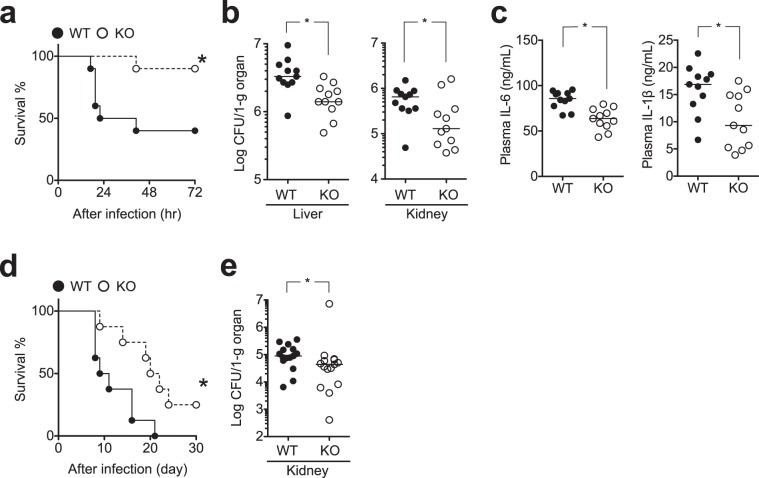
LMIR3-KO mice are more resistant to bacterial and fungal infections. Mice were injected intraperitoneally and intravenously with *P*. *aeruginosa* PAO1 (**a**–**c**) and *C*. *albicans* SC5314 (**d**,**e**), respectively (**a**, 6 × 10^4^ cells/mouse, n = 10; (**b**,**c**), 5 × 10^6^ cells/mouse, n = 11; (**d**) 5 × 10^4^ cells/mouse, n = 8; and e, 2–3 × 10^5^ cells/mouse, n = 15). The survival rate (**a**,**d**), plasma cytokine level (**c**), and microbial burden in the organs (**b**, at 3 h post infection and (**e**) at 3 days post infection) were measured. Representative survival curves from three independent experiments, which were compared using log-rank tests, are shown. Pooled data of the microbial burden from 2–3 independent experiments are plotted, and median values are shown with short horizontal lines. These were evaluated using the Mann–Whitney U test. *P < 0.05 versus WT.

We next validated the above evidence using mouse infection models for *Pseudomonas* peritonitis^37,38^ and disseminated candidiasis^39^. The resolution of these infections *in vivo* is strongly dependent on neutrophil microbicidal activity^40,41^. In both infection models, LMIR3-KO mice had significantly improved survival rates (Fig. 7a,d) and reduced microbial burden in their organs (Fig. 7b,e). The numbers of neutrophils in the liver and peritoneal lavage of WT and LMIR3-KO mice were comparable 3 h after *P*. *aeruginosa* infection (Supplementary Fig. 4). Excessive production of inflammatory cytokines, including IL-6 and IL-1β, was not found in the peripheral blood of LMIR3-KO mice (Fig. 7c). These results suggested that LMIR3 deficiency led to resistance against *Pseudomonas* peritonitis and disseminated candidiasis.

## Discussion

In this study, we demonstrated that LMIR3 deficiency led to enhanced antimicrobial properties in neutrophils and host resistance against lethal bacterial and fungal infections.

In this study, we mainly focused on the function of LMIR3 on mouse neutrophils; however, it is also important to elucidate the function of hLMIR3 in neutrophils. We observed hLMIR3 expression in HL-60 cells (Supplementary Fig. 1c). Although a previous report demonstrated that human peripheral granulocytes express hLMIR3^42^, it remains to be elucidated whether immature BM progenitors express hLMIR3, and whether hLMIR3 can regulate neutrophil antimicrobial activity.

In the present study, HOCl production and elastase activity or releasability were significantly increased in LMIR3-KO neutrophils (Fig. 4a,b, and Supplementary Fig. 3b). However, MPO expression was comparable between WT and LMIR3-KO neutrophils (Fig. 5c). Although the number of primary granules and membrane protein expression were slightly increased in LMIR3-KO neutrophils (Fig. 5a,b,d), these minor changes may not be the principal reason for these enhanced antimicrobial properties. MPO expression was comparable between WT and LMIR3-KO neutrophils, but HOCl production was increased in LMIR3-KO neutrophils: suggesting that MPO activity is upregulated in LMIR3-KO CD11b^high^ Ly6G^+^ neutrophils. To address the mechanism by which LMIR3 regulates MPO activity, the absolute amount of ROS in primary granules, including HOCl as well as H_2_O_2_ and O_2_^−^, should be determined in a future study. The possibility that LMIR3 can regulate the enzymatic activities of MPO, elastase, and other proteases is also an important subject for future studies. There is a possibility that LMIR3 regulates the production of antimicrobial molecules and their release via inhibitory signals. Future studies are required to elucidate the molecular mechanism of the inhibitory signals in neutrophils.

It is known that the elastase and MPO-catalysed HOCl in primary granules are required to kill bacterial and fungal pathogens, including *P*. *aeruginosa* and *C*. *albicans*^20,30–33^. LMIR3-KO neutrophils displayed significantly augmented antimicrobial activity *in vitro*, whereas elastase and MPO inhibitors offset this enhanced antimicrobial activity in LMIR3-KO neutrophils (Fig. 6). These findings suggested that the enhanced production of elastase and HOCl increased the antimicrobial activity of LMIR3-KO neutrophils. However, these inhibitors did not entirely eliminate the antimicrobial activity of LMIR3-KO neutrophils (Fig. 6b); thus, other pathways conferring antimicrobial activity may also be upregulated in LMIR3-KO neutrophils. Future studies are also required to investigate the antimicrobial molecules conferring the enhanced antimicrobial activity of LMIR3-KO neutrophils.

In this study, we also demonstrated that LMIR3-KO mice had significantly enhanced host resistance to lethal bacterial and fungal infection without excessive inflammatory response (Fig. 7). Previous studies indicated that neutrophil serine proteases participate in both innate immunity via microbial killing and initiation of acquired immunity by stimulating lymphocytes. Furthermore, neutrophil serine proteases play a role in the fine-tuning of inflammatory responses by digesting cytokines, chemokines, and surface receptors^43^. Thus, it is likely that LMIR3-deficient neutrophils are involved in LMIR3-KO-related host resistance to microbial infection. However, previous studies demonstrated that eosinophils^12^, mast cells^11^, MΦs^8,44,45^, and DCs^46^ also express surface LMIR3; hence, the antimicrobial activity of these cells may also be boosted in LMIR3-KO mice after *in vivo* infection, and these myeloid cells may also be involved in the host resistance to *Pseudomonas* peritonitis and disseminated candidiasis. Future studies are required to evaluate the mechanism by which neutrophils contribute to host resistance in LMIR3-KO mice after infection.

A recent study uncovered that LMIR3 deficiency leads to increased survival in a CLP model through the increased recruitment of neutrophils to the peritoneal cavity in a mast cell-dependent manner^6^. In the CLP model, neutrophil chemoattractant production in the peritoneal cavity was increased by LMIR3 deficiency, whereas the intrinsic migratory activity of neutrophils remained unaltered^6^. By contrast, we observed comparable accumulation of inflammatory cells, including neutrophils, in the peritoneal cavity and liver of WT and LMIR3-KO mice 3 h after *P*. *aeruginosa* infection (Supplementary Fig. 4). Although the reasons for the difference in LMIR3-KO neutrophil accumulation between the CLP study and our study remain unknown, several factors may be involved, such as the different types of microbes (endogenous gut-derived polymicrobial infection in the CLP model vs *P*. *aeruginosa* infection in our study), the different number of microbes, and the presence or absence of CLP.

In conclusion, the present study is the first to demonstrate that mouse LMIR3 deficiency enhances the antimicrobial ability of neutrophils. These findings may facilitate the development of new treatments for clinically important bacterial and fungal infections.

## Methods

### Ethical statement

All animal experiments were approved by the ethical committee of the National Institute of Infectious Disease, Japan (approval numbers 117162, 213067, 214035, 213068, 214038, 214030, 215035, 215032, and 215037) and were performed in accordance with the approved guidelines and regulations.

### Mice

C57BL/6J mice were purchased from Japan SLC, Inc. LMIR3-KO mice were generated in a previous study^3^. TLR4-KO and MyD88-KO mice were purchased from Oriental Bio Service, Inc. All KO mice were of the C57BL/6 J background. Mice used in experiments were sex-matched and age-matched (7–16 weeks old) and were maintained under specific-pathogen-free conditions at the National Institute of Infectious Diseases of Japan.

### Microbes

The *P*. *aeruginosa* strain PAO1, *E*. *coli* JM109, *Streptococcus pneumoniae* strain WU2, and *C*. *albicans* strain SC5314 strains were cultured in tryptic soy broth, Luria–Bertani broth, Todd–Hewitt broth supplemented with 0.5% (w/v) yeast extract, and yeast extract–peptone–dextrose (YPD) broth, respectively. These powder media were purchased from BD Biosciences. To prepare heat-inactivated microbes, stationary phase cultures of *P*. *aeruginosa* (37 °C), *E*. *coli* (37 °C), and *C*. *albicans* (30 °C), and an exponential phase cultures of *S*. *pneumoniae* (37 °C, 5% CO_2_) were harvested, washed with Dulbecco’s phosphate-buffered saline (DPBS; Gibco) and resuspended in DPBS. To determine the number of live microbes present, cell suspensions were spread onto agar plates containing the respective aforementioned media before heat-inactivation. These plates were incubated for 24 h at the respective aforementioned temperatures, and the colonies were subsequently counted. For microbial inactivation, cell suspensions were boiled for 1 h. Heat-inactivated microbes were not washed further. To ensure complete inactivation, boiled suspensions were also plated onto the respective media respectively and incubated for 7 days.

### Reagents

TLR ligands (ApoTech) and TLRgrade™ LPS from *E*. *coli* serotype O55:B5 (Enzo Life Science, Inc.) were used (Fig. 2). In addition, polymyxin B (Wako) and APDC (Dojindo) were dissolved in water and filter-sterilised. For electron microscopy, DAB and 3% (v/v) hydrogen peroxide (Muto Pure Chemicals) were used. To inhibit neutrophil elastase and MPO, Sivelestat and 4-ABAH were purchased from Cayman and used for the neutrophil inhibition assay as described above.

### Cell isolation

BM cells were harvested from the femurs and tibiae of mice. Erythrocytes were lysed with lysis buffer [15 mM ammonium chloride, 1 mM sodium bicarbonate, and 100 mM ethylenediaminetetraacetic acid (EDTA)/disodium, pH 7.4]. The cells were washed with RPMI 1640 medium to remove residual lysis buffer, and BM cells directly used in the subsequent experiments. To enrich for neutrophils, the BM cells were suspended in DPBS without lysing the red blood cells and layered onto Histopaque^®^1077 and Histopaque^®^1119 (Sigma). The cells were centrifuged at 700 × *g* without acceleration and deceleration for 30 min at room temperature. The erythrocytes were precipitated at the bottom of the tube, and. the granulocytes at the interface of Histopaque^®^1077 and Histopaque^®^1119 were collected and washed twice with RPMI 1640 medium supplemented with 10% (w/v) foetal bovine serum (FBS). Ly6G^+^ cells were enriched using anti-Ly6G mAb (1A8, phycoerythrin-labelled, BioLegend), anti-phycoerythrin MicroBeads, and a MACS® Cell separator (Miltenyi Biotec) according to the manufacturer’s instructions.

### Differentiation of HL-60 cells

HL-60 cells (2 × 10^5^ cells/mL) were differentiated for 7 days in RPMI 1640 medium supplemented with 10% (w/v) FBS and 1.25% (v/v) DMSO at 37 °C and 5% CO_2_. The surface expression of hLMIR3 and CD11b was measured via flow cytometry as described below.

### Flow cytometry

To evaluate the expression levels of LMIR3 and other markers (listed below), BM cells with/without cultivation (18 h) were suspended in FACS buffer [DPBS containing 2 mM EDTA, 0.5% (w/v) bovine serum albumin, and 0.1% (w/v) sodium azide] and stained with antibodies for CD45 (30-F11), CD11b (M1/70), Ly6G (1A8), c-kit (2B8), CD34 (HM34), LMIR3 (3-14-11), hLMIR3(UP-D2), CD115 (AFS98), CD63 (NVG-2), MPO (2D4, Abcam), and CD68 (FA-11). The LMIR3-specific mAb (clone 3-14-11) was developed in a previous study^3^. Dead cells and apoptotic cells were stained using propidium iodide and annexin-V, respectively. Cell staining was performed after blocking Fc receptors using an anti-mouse CD16/32 mAb (clone: 93) or human BD Fc block™ (BD Bioscience). After surface staining and fixation with DPBS containing 4% (w/v) paraformaldehyde, the intracellular proteins were stained with permeabilisation buffer according to the manufacturer’s instructions. To distinguish intracellular CD68 from extracellular CD68, a different fluorophore was selected for this step. Unless otherwise noted, antibodies and buffers were purchased from BioLegend.

To evaluate HOCl production, BM cells were incubated with 2.5 μM HySOx (Goryo Chemical, Inc.), a specific fluorescent probe for HOCl, and its fluorescent signal was evaluated in the phycoerythrin^21–23^. Subsequently, HySOx-stained-BM cells were labelled with fluorescent antibodies to evaluate the neutrophil cell-surface markers, as described above, without fixation. Finally, all cells were suspended in FACS buffer and analysed via flow cytometry. Data acquisition and cell sorting were performed using a BD FACSCalibur, BD FACSCanto II, or BD FACSAria III flow cytometer (BD Bioscience) and analysed using FlowJo software (Tree Star, Inc.).

### Immunoblotting

Sample preparation and immunoblotting analysis were performed as described previously^47^. Adjusted number of BM cells, cultured BM cells, and sorted cells were washed with DPBS, and fixed for 60 min with 10% (w/v) trichloroacetic acid. The fixed cells (1 × 10^6^ cells) were harvested by centrifugation for 5 min at 320 × *g*, and lysed using 20 μL of 9 M urea containing 2% (v/v) Triton X-100 and 5 μL of 10% (w/v) lithium dodecyl sulphate. The lysates were treated with 1-μL of 1,4-dithiothreitol (DTT)/bromophenol blue (BPB) mixed solution [100 μL of 1 M DTT and 50 μL of 4% (w/v) BPB] to reduce the protein disulphide bonds and neutralised with 1 μL of 2 M Tris solution, yielding a clear blue final protein solution. The proteins were separated via SDS-PAGE (constant 200 V, 30 min) using 10% precast polyacrylamide gel (BioRad) and Tris–glycine–SDS buffer (25 mM Tris, 192 mM glycine, and 0.1% SDS). The proteins were then transferred to a 0.2-μm Immun-Blot^®^ PVDF membrane (BioRad) via a Trans-Blot^®^ SD Semi-Dry Transfer Cell (constant 15 V, 30 min; BioRad) using Towbin transfer buffer [25 mM Tris, 192 mM glycine, and 20% (v/v) methanol]. The membrane was blocked with PVDF Blocking Reagent (Toyobo) and treated with antibody solution using *Can Get Signal*^®^ Immunoreaction Enhancer Solution (Toyobo). Anti-mouse LMIR3 goat polyclonal IgG (1:1000 dilution; AF2788, R&D), bovine anti-goat IgG (H + L) peroxidase-AffiniPure (1:10,000 dilution; 805-035-180, Jackson ImmunResearch), anti-mouse α-tubulin rabbit polyclonal antibody (1:5000 dilution; 2144 S, Cell Signaling Technology), and goat anti-rabbit IgG (H + L) peroxidase-AffiniPure (1:10,000 dilution; 111-035-003, Jackson ImmunResearch) were used to detect each target protein. Precision Plus Protein™ WesternC™ Standards (3 μL/lane; BioRad) and Precision Protein™ StrepTactin-HRP Conjugate (1:200,000 dilution; BioRad) were used to monitor the protein molecular mass. For the loading control and re-probing, antibody-stripping was performed using the general acid–glycine method [0.2 M glycine, 0.1% (w/v) SDS, and 1% (v/v) Tween-20, adjusted to pH 2.2]^48^. Immobilon™ western chemiluminescent HRP substrate (Merck) and C-DiGit^®^ blot scanner (M&S TechnoSystems) were used for band detection.

### Elastase

BM cells (1 × 10^6^ cells) and heat-inactivated *P*. *aeruginosa* [1 × 10^7^ cells, multiplicity of infection (MOI) = 10] in 200 μL of RPMI 1640 medium without serum were added to 96-well plates. The cells were incubated for 2 h at 37 °C and 5% CO_2_, and the culture supernatants were collected after centrifugation at 320 × *g* for 5 min. Elastase in the supernatant was measured with an Enzchek^®^ elastase assay kit (Thermo Fisher Scientific). The fluorescent elastin substrate was incubated in the culture supernatant for 1 h, and the fluorescent intensity was subsequently measured using a DTX-880 microplate reader (Beckman Coulter) set for excitation at 480 nm and emission detection at 520 nm. The fluorescent intensity of the blank control was subtracted from that of the samples, and the resulting value was multiplied by a coefficient value (1e-04) for conversion in arbitrary units (AUs). The positive control, namely 1 unit/mL of pig pancreatic elastase, had an approximate value of 4,000 AUs in our assays.

### Transmission electron microscopy

To enhance the electron density of peroxidase-containing primary granules^49^, Histopaque-enriched neutrophils were incubated in DPBS containing 1 mg/mL DAB and 0.15% (v/v) hydrogen peroxide for 5 min at room temperature. The stained cells were washed twice with DPBS and fixed in 0.1 M sodium cacodylate buffer containing 2.5% (v/v) glutaraldehyde and 2% (w/v) paraformaldehyde at 4 °C. The cells were post-fixed in 1% (w/v) osmium tetroxide, and embedded in 2% (w/v) ultrapure agarose. After block staining with 1% aqueous uranyl acetate and serial dehydration, the samples were embedded in Epon resin. Ultrathin sections were mounted on copper-coated grids and post-stained with saturated uranyl acetate and lead citrate. The sections were observed using an HT7700 transmission electron microscope (Hitachi High Technologies).

### Neutrophil-mediated microbicidal effect

*P*. *aeruginosa* and *C*. *albicans* at stationary phase (MOI = 0.1) were co-cultured in 96-well plates in RPMI medium containing 10% (w/v) FBS with/without Histopaque-enriched neutrophils (0.2–1 × 10^6^ cells/well). After a 1-h incubation at 37 °C and 5% CO_2_, 200 μL of *P*. *aeruginosa* and neutrophil suspensions were transferred to 1.5-mL microtubes. The wells were rinsed with 800-μL of sterile distilled water, and the lavage fluid was also transferred to the same 1.5-mL tubes. The suspensions were vigorously mixed to lyse the neutrophils, and the lysate was spread onto the tryptic soy agar. These plates were incubated for 1 day at 37 °C, and colonies were counted to determine the number of colony-forming units (CFU). Because *C*. *albicans* forms hyphae and tightly adheres to the wells under the aforementioned condition, the optical density in the wells was measured at 590 nm (OD_590_) using a DTX-880 microplate reader (Beckman Coulter) to evaluate the *C*. *albicans* growth level after 18 h of cultivation. To establish phagocytosis-based killing, the 96-well plate was centrifuged for 10 min at 700 × *g* at room temperature to precipitate the neutrophils and microorganisms. To inhibit neutrophil elastase and MPO, 25 μg/mL Sivelestat and 4-ABAH were added to the medium during cultivation.

### Infection study

Cultures of *P*. *aeruginosa* in log phase and *C*. *albicans* in stationary phase were harvested, washed twice with DPBS, and resuspended in DPBS. The OD_600_ was measured to determine the bacterial concentration, and the bacterial suspension was adjusted with DPBS to 3 × 10^5^ and 2.5 × 10^7^ cells/mL for the survival experiment and measurement of bacterial burden in organs, respectively. Viable yeast cells were enumerated using trypan blue staining. The fungal suspension was also adjusted with DPBS to 2.5 × 10^5^ and 1–1.5 × 10^6^ cells/mL for the survival experiment and measurement of fungal burden in organs, respectively. Bacterial and yeast suspensions (200 μL) were injected into mice intraperitoneally and intravenously, respectively. The mice were euthanised via carbon dioxide inhalation, and their organs were dissected and transferred to DPBS to determine weight and microbial burden. The organs were homogenised using a 70-μm cell strainer (BD Biosciences), and the homogenates were diluted and plated onto the tryptic soy agar for *P*. *aeruginosa* and YPD plates for *C*. *albicans*, followed by overnight incubation at 37 °C and 30 °C, respectively. The colonies were counted after incubation.

### Cytokine measurement

The cytokine levels of heparinised plasma specimens were determined using enzyme-linked immunosorbent assay (ELISA). A MaxiSorp plate (Nunc) and a DuoSet ELISA kit (R&D Systems) or BD OptEIA ELISA sets (BD Bioscience) were used according to the manufacturers’ instructions.

### Statistical analysis

GraphPad Prism5 (GraphPad Software, Inc.) was used for statistical analyses, and the analysis depended upon the data collected (please see the Results section). *P* values less than 0.05 were considered significant.

## Electronic supplementary material

Fig. S1-S4

## Data Availability

The datasets generated during the current study are available from the corresponding author on reasonable request.
