# Supplementary material for: Mouse LIMR3/CD300f is a negative regulator of the antimicrobial activity of neutrophils
Source: Sci Rep. 2018 Nov 27;8:17406. doi: 10.1038/s41598-018-35699-4 (PMC6258681; doi:10.1038/s41598-018-35699-4)
Supplement: Supplementary file 1 — Fig. S1-S4 [file 41598_2018_35699_MOESM1_ESM.pdf]

Supplementary information

Mouse LIMR3/CD300f is a negative regulator of the antimicrobial activity of neutrophils

Keigo Ueno[1], Makoto Urai[1†], Kumi Izawa[2], Yoshiko Otani[1,3], Nao Yanagihara[1,3], Michiyo Kataoka[4], Shogo Takatsuka[1], Masahiro Abe[1], Hideki Hasegawa[4], Kiminori Shimizu[3], Toshio Kitamura[5], Jiro Kitaura[2], Yoshitsugu Miyazaki[1], and Yuki Kinjo[1*††]

1. Department of Chemotherapy and Mycoses, National Institute of Infectious Diseases, 1-23-1 Toyama, Shinjuku-ku, Tokyo 162-8640, Japan
2. Atopy Research Center, Juntendo University School of Medicine, 2-1-1 Hongo, Bunkyo-ku, Tokyo 113-8421, Japan
3. Department of Biological Science and Technology, Faculty of Industrial Science and Technology, Tokyo University of Science, 6-3-1 Niijuku, Katsushika-ku, Tokyo 125-8585, Japan
4. Department of Pathology, National Institute of Infectious Diseases, 1-23-1 Toyama, Shinjuku-ku, Tokyo 162-8640, Japan
5. Division of Cellular Therapy, Advanced Clinical Research Center, The Institute of Medical Science, The University of Tokyo; Division of Stem Cell Signaling, The Institute of Medical Science, The University of Tokyo, 4-6-1 Shirokanedai, Minato-ku, Tokyo 108-8639, Japan

† Present address: Department of Chemistry for Life Sciences and Agriculture, Faculty of Life Sciences, Tokyo University of Agriculture, 1-1-1 Sakuragaoka, Setagaya-ku, Tokyo 156-8502, Japan

†† Present address: Department of Bacteriology, The Jikei University School of Medicine, 3-25-8 Nishi-Shimbashi, Minato-ku, Tokyo 105-8461, Japan

* Address correspondence to Yuki Kinjo, ykinjo@niid.go.jp

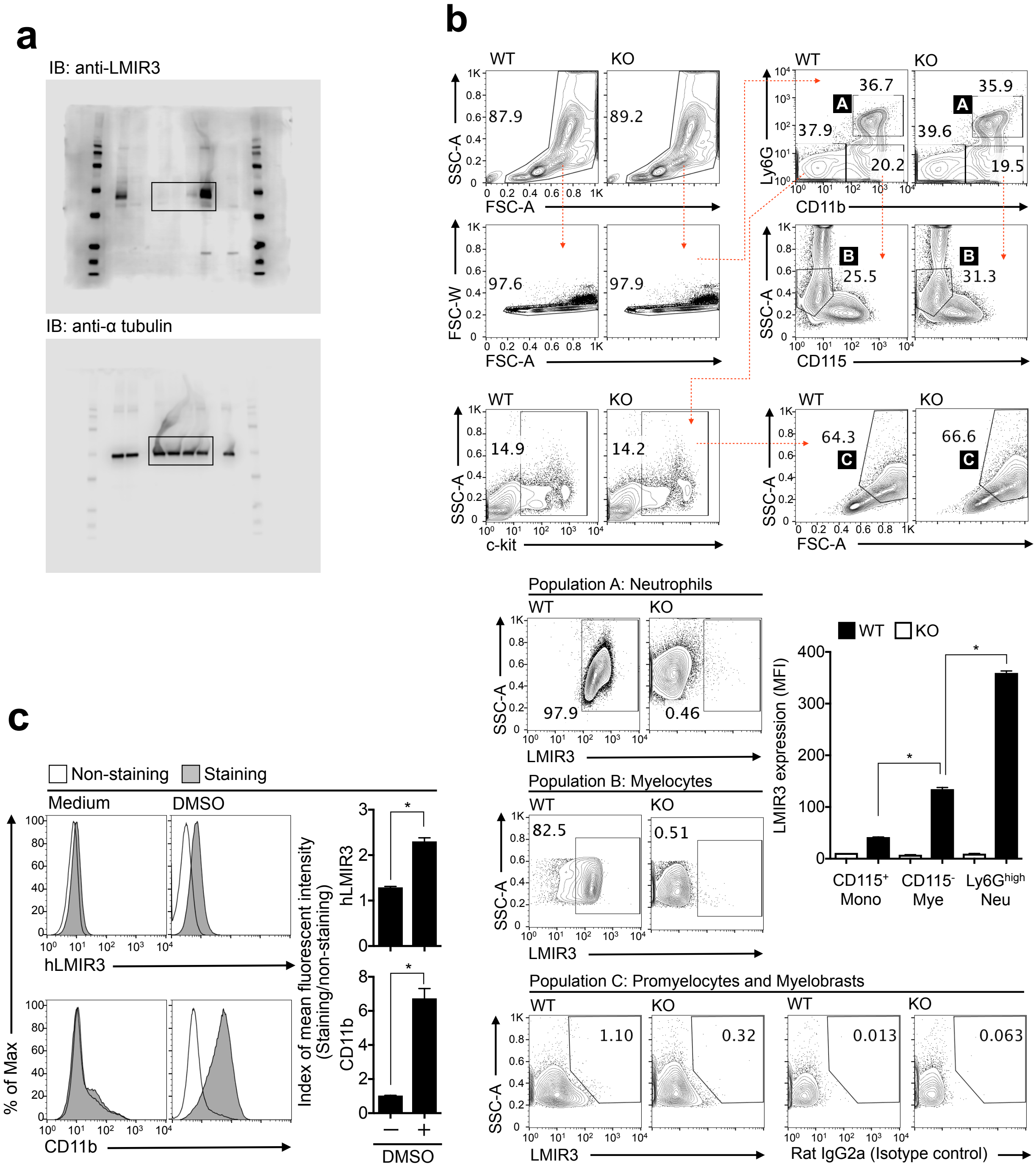

Supplementary Figure 1. LMIR3 is expressed on mouse and human neutrophils. (a) Full-length blots of main Figure 1a are shown. Boxes indicate the cropped regions. (b) LMIR3-expressing neutrophils and immature neutrophils. BM cells were analysed as described in Figure 1b using the gating strategy as depicted. Representative data (mean ± SDs, n = 3) from three independent experiments are shown. Mono, monocytes; Mye, myelocytes; Neu, Neutrophils; *, $P < 0.05$ as determined using analysis of variance with a Tukey's *post hoc* test. (c) HL-60 cells were differentiated via exposure to 1.25% (v/v) DMSO for 7 days, and the surface hLMIR3 expression was evaluated. Representative data (mean ± SDs, n = 3) from four independent experiments are shown. *, $P < 0.05$ as determined using an unpaired t-test.

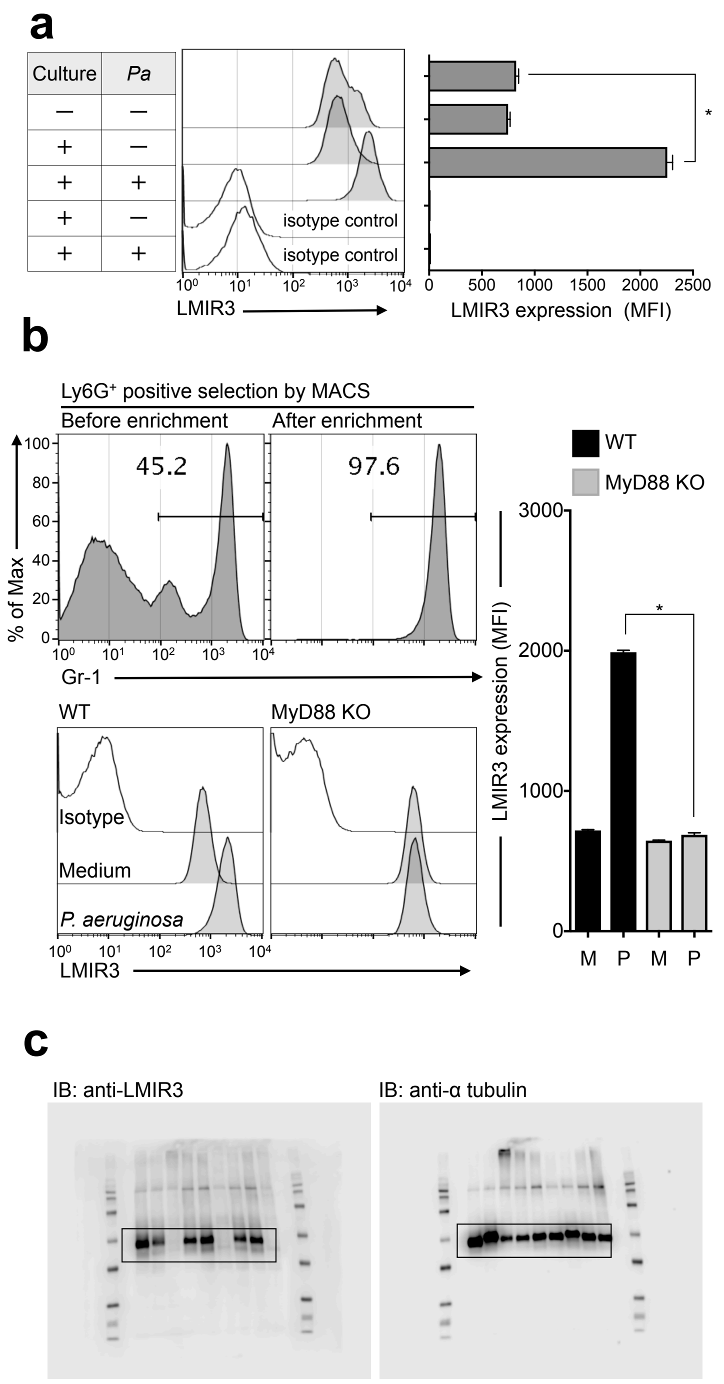

Supplementary Figure 2. Surface LMIR3 expression on neutrophils is further increased by bacterial stimulation.

LMIR3 expression was measured as described in Figure 2. For the bacterial stimulation, heat-inactivated *P. aeruginosa* (*Pa*, MOI = 1) was used. The flow cytometry gate was set for CD11b⁺ Ly6G⁺ neutrophils to compare LMIR3 expression before and after the cultivation (a). Ly6G⁺ cells were enriched using magnetic-activated cell sorting and then analysed as described in Figure 2. M, Medium; P, Heat-inactivated *P. aeruginosa* MOI = 1 (b). Representative data (mean ± SDs) from two independent experiments are shown. *, $P < 0.05$ as determined using an unpaired *t*-test (a–b). Full-length blots from main Figure 3 are shown. Boxes indicated the cropped regions (c).

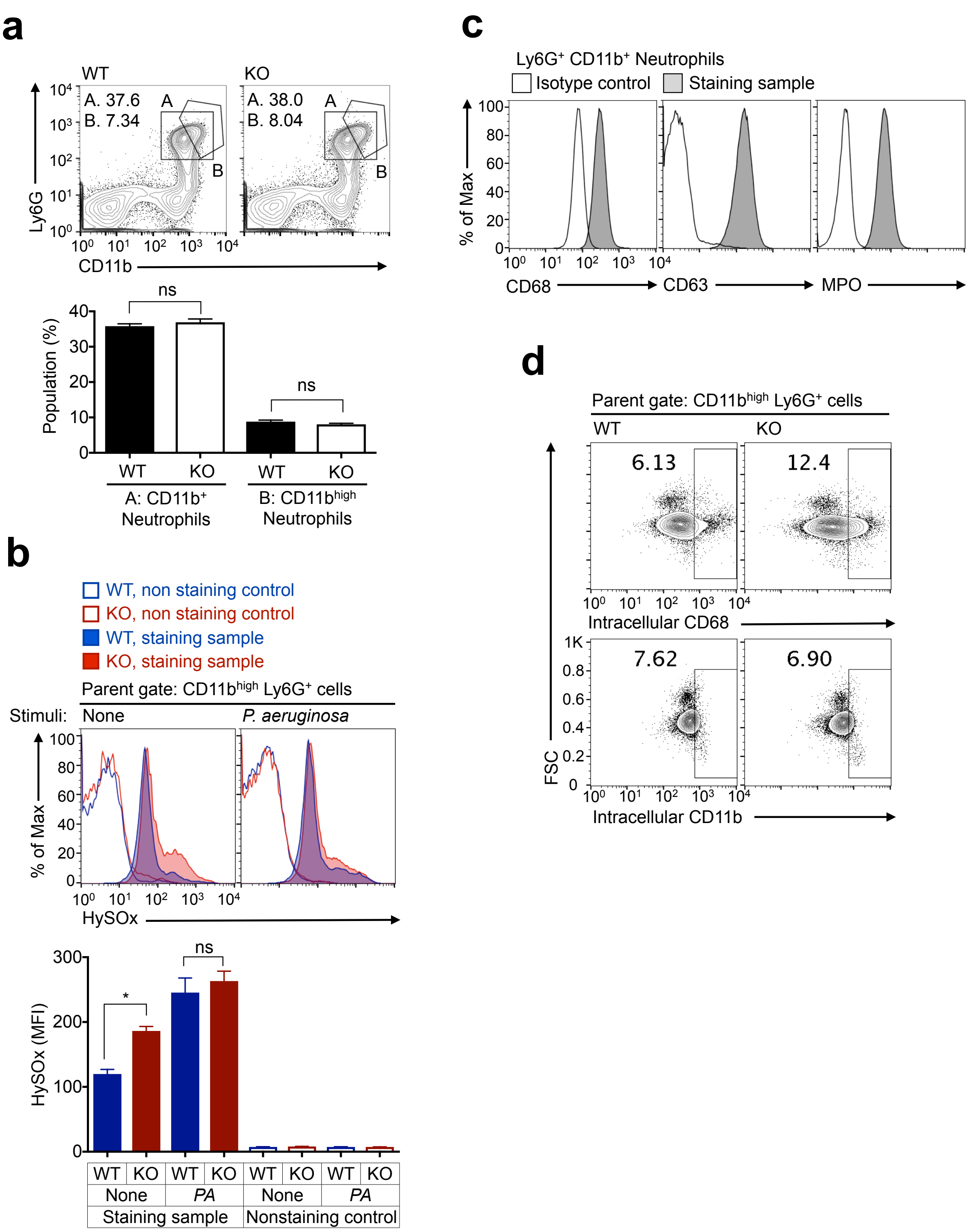

Supplementary Figure 3. Percentage of BM neutrophils and marker profiles.

(a) The comparable percentage of BM neutrophils between WT and LMIR3-KO mice is shown. BM cells were harvested from WT and LMIR3-KO mice and analysed via flow cytometry. The gates were set for $CD11b^{+}$ $Ly6G^{+}$ neutrophils and $CD11b^{high}$ $Ly6G^{+}$ neutrophils. (b) HOCl production was evaluated using MFI value of HySOx staining. BM cells were analysed as described in Figure 4. *PA*: *P. aeruginosa.* (a–b) Pooled data from two independent experiments are shown (mean ± SEMs, n = 8). (c) The expression levels of the intracellular CD68, CD63, and MPO were analysed as described in Figure 5a–c and compared with those of the isotype control. All neutrophils clearly expressed

these proteins. (d) The expression profile of intracellular CD11b in WT and KO neutrophils. The gates were set for CD11b^{high} Ly6G^{+} neutrophils as described in Supplementary Figure 3a. The expression profile was analysed as described in Figure 5a–c. In contrast to CD68 and CD63, CD11b is known to be localised on the membranes of specific granules, gelatinase granules, and secretory vesicles.

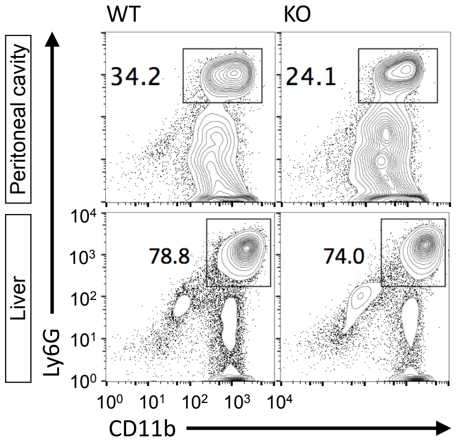

Supplementary Figure 4. Neutrophil counts were not increased in LMIR3-KO mice following *P. aeruginosa* infection. Infection challenge was performed as described in Figure 7. At 3 h post infection, the leukocytes were harvested from peritoneal lavage and collagenase D-treated livers. Representative profiles from three independent experiments are shown.
